# Purinergic agonists increase [Ca^2+^]_i_ in rat conjunctival goblet cells through ryanodine receptor type 3

**DOI:** 10.1152/ajpcell.00291.2024

**Published:** 2024-08-05

**Authors:** Haakon K. Fjaervoll, Ketil A. Fjaervoll, Menglu Yang, Ole K. Reiten, Jeffrey Bair, Changrim Lee, Tor P. Utheim, Darlene Dartt

**Affiliations:** ^1^Division of Head, Neck and Reconstructive Surgery, Faculty of Medicine, Institute of Clinical Medicine, University of Oslo, Oslo, Norway; ^2^Department of Ophthalmology, Schepens Eye Research Institute, Massachusetts Eye and Ear Infirmary, Harvard Medical School, Boston, Massachusetts, United States; ^3^Department of Medical Biochemistry, Oslo University Hospital, Oslo, Norway; ^4^Department of Plastic and Reconstructive Surgery, Oslo University Hospital, Oslo, Norway

**Keywords:** ATP, BzATP, calcium homeostasis, conjunctival goblet cells, ryanodine receptor 3

## Abstract

ATP and benzoylbenzoyl-ATP (BzATP) increase free cytosolic Ca^2+^ concentration ([Ca^2+^]_i_) in conjunctival goblet cells (CGCs) resulting in mucin secretion. The purpose of this study was to investigate the source of the Ca^2+^_i_ mobilized by ATP and BzATP. First-passage cultured rat CGCs were incubated with Fura-2/AM, and [Ca^2+^]_i_ was measured under several conditions with ATP and BzATP stimulation. The following conditions were used: *1*) preincubation with the Ca^2+^ chelator EGTA, *2*) preincubation with the SERCA inhibitor thapsigargin (10^−6^ M), which depletes ER Ca^2+^ stores, *3*) preincubation with phospholipase C (PLC) or protein kinase A (PKA) inhibitor, or *4*) preincubation with the voltage-gated calcium channel antagonist nifedipine (10^−5^ M) and the ryanodine receptor (RyR) antagonist dantrolene (10^−5^ M). Immunofluorescence microscopy (IF) and quantitative reverse transcription polymerase chain reaction (RT-qPCR) were used to investigate RyR presence in rat and human CGCs. ATP-stimulated peak [Ca^2+^]_i_ was significantly lower after chelating Ca^2+^_i_ with 2 mM EGTA in Ca^2+^-free buffer. The peak [Ca^2+^]_i_ increase in CGCs preincubated with thapsigargin, the PKA inhibitor H89, nifedipine, and dantrolene, but not the PLC inhibitor, was reduced for ATP at 10^−5^ M and BzATP at 10^−4^ M. Incubating CGCs with dantrolene alone decreased [Ca^2+^]_i_ and induced CGC cell death at a high concentration. RyR3 was detected in rat and human CGCs with IF and RT-qPCR. We conclude that ATP- and BzATP-induced Ca^2+^_i_ increases originate from the ER and that RyR3 may be an essential regulator of CGC [Ca^2+^]_i_. This study contributes to the understanding of diseases arising from defective Ca^2+^ signaling in nonexcitable cells.

**NEW & NOTEWORTHY** ATP and benzoylbenzoyl-ATP (BzATP) induce mucin secretion through an increase in free cytosolic calcium concentration ([Ca^2+^]_i_) in conjunctival goblet cells (CGCs). The mechanisms through which ATP and BzATP increase [Ca^2+^]_i_ in CGCs are unclear. Ryanodine receptors (RyRs) are fundamental in [Ca^2+^]_i_ regulation in excitable cells. Herein, we find that ATP and BzATP increase [Ca^2+^]_i_ through the activation of protein kinase A, voltage-gated calcium channels, and RyRs, and that RyRs are crucial for nonexcitable CGCs’ Ca^2+^_i_ homeostasis.

## Graphical abstract

**Figure:**
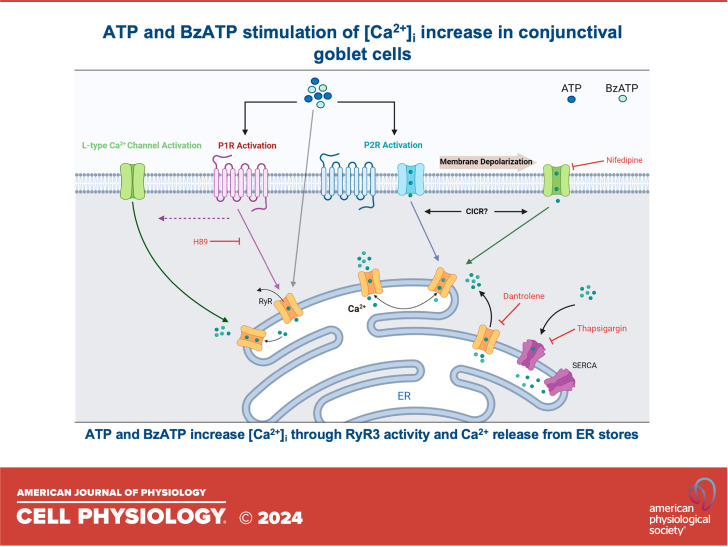

## INTRODUCTION

Mucins are high molecular weight glycosylated proteins that lubricate the surface of the cornea and hinder foreign particles from binding to the ocular surface epithelium. The large gel-forming mucin MUC5AC is a particularly important mucin in tears because of its high relative quantity to other gel-forming mucins and is secreted by conjunctival goblet cells (CGCs) (1). Ocular surface inflammation is associated with goblet cell loss and impaired mucin secretion (2, 3). Current research focuses on investigating how to restore these goblet cells and their secretion of MUC5AC in patients with ocular surface disease, such as dry eye disease (4–6).

Ionized calcium (Ca^2+^) is an important cellular messenger involved in a variety of cellular functions, including muscle contraction, exocytosis, gene expression, and cell proliferation (7–10). Regulation of free cytosolic calcium concentrations ([Ca^2+^]_i_) is complex and strictly regulated (11). Calcium ions are able to enter cells through various channels located on the cell membrane, such as voltage-gated and receptor-operated calcium channels (VGCCs and ROCCs) (12). In addition, the [Ca^2+^]_i_ is regulated by intracellular calcium stores, including the endoplasmic reticulum (ER), which can sequester and release calcium ions as needed (13). Once plasma membrane receptors that are coupled to phospholipase C (PLC) are activated, inositol 3-phosphate (IP3) is released to the cytosol and activates IP3 receptors on the ER. The result is the efflux of Ca^2+^ through the ER membrane, which is a common mechanism in nonexcitable cells to increase [Ca^2+^]_i_ (14). Ryanodine receptors (RyRs) are large (2,200 kDa) calcium channels located on the sarcoplasmic/endoplasmic reticulum of cells. They are regulated by a number of ions and active molecules, including Ca^2+^, Mg^2+^, ATP, protein kinase A (PKA), cyclic ADP-ribose (cADPr), and calmodulin among others (15–17). RyRs have been mostly studied in excitable muscle and neuronal cells. RyR1 is predominantly described in striated muscle cells together with VGCCs, RyR2 in cardiomyocytes, and RyR3 in neurons and glial cells (16, 18). In these cells, RyRs exhibit pivotal functions in the regulation of [Ca^2+^]_i_. Small changes in the intracellular concentration of Ca^2+^ lead to physiological or pathological processes in cells and tissues (19, 20). An increase in [Ca^2+^]_i_ in CGCs usually results in mucin secretion through exocytosis (21).

Together with Ca^2+^, ATP is an important molecule that acts both in energy storage and as a signaling molecule in normal and pathological processes (11, 22). In inflammation and infection, ATP is considered a damage-associated molecular pattern (DAMP) molecule and can function via purinergic receptors to activate inflammasomes (23–25). After ATP is released into the extracellular environment through multiple types of lytic or nonlytic mechanisms, it can stimulate the ionotropic purinergic type 2 X (P2X; P2X1-7) and metabotropic purinergic type 2 Y (P2Y; P2Y1, P2Y2, P2Y4, P2Y6 and P2Y11-14) receptors on adjacent cells, or be degraded to ADP, AMP, and adenosine by ectonucleotidases (CD39 and CD73) (26). These metabolites of ATP can then activate both P2Y and purinergic type 1 (P1) receptors (27). P2Y receptors (P2YRs) are G-protein coupled receptors (GPCRs) that couple to G-proteins, primarily Gq, which signals through PLC and IP3 receptors on the ER (28). All three IP3 receptors are present on CGCs (29). P1 (A1, A2A, A2B, and A3) adenosine receptors (ARs) also couple to G-proteins of which A2AAR and A2BAR couple mainly to Gs and increase cAMP, whereas A1AR and A3AR couple to Gi and decrease cAMP (26, 30, 31).

Previous work from our laboratory showed that ATP and its analog BzATP induce mucin secretion in rat CGCs (32). However, the signaling mechanisms through which ATP and BzATP increase [Ca^2+^]_i_ and induce mucin secretion in CGCs are largely unclear. The aim of the present study is, therefore, to investigate which signaling pathway(s) is used by ATP and BzATP to increase [Ca^2+^]_i_.

## MATERIALS AND METHODS

### Animals

All animal usage abided by the ARVO Statement for the Use of Animals in Ophthalmic and Vision Research and was approved by the Schepens Eye Research Institute Animal Care and Use Committee (IACUC). Four- to eight-week-old male Sprague Dawley rats were obtained from Taconic Farms (Germantown, NY) and euthanized with CO_2_ for 5–7 min. The bilateral conjunctivae were removed.

### Human Materials

Human conjunctival tissue was obtained from Eversight Eye Bank (Ann Arbor, MI). Conjunctival tissue was placed in Optisol-GS media within 6 h of death. Use of this tissue was reviewed by the Massachusetts Eye and Ear Human Studies Committee and was considered to not meet the definition of research with human subjects. Therefore, no further approvals were required. Human tissue was only used to determine the presence of RyRs in CGCs with quantitative reverse transcription polymerase chain reaction on mRNA and immunofluorescence microscopy.

### Cell Culture

Goblet cells were cultured from rat or human conjunctival explants as described previously (33, 34). Conjunctival epithelial pieces were placed in 6-well plates and cultured in RPMI 1640 medium supplemented with 10% fetal bovine serum, 2 mM glutamine, and 100 mg/mL penicillin-streptomycin for 7–9 days for rat cells and 14 days for human cells. First-passage CGCs were used in all experiments.

### Immunofluorescence Microscopy

Immunofluorescence (IF) microscopy was performed as previously described, with minor modifications (33). First-passage CGCs were cultured on coverslips and fixed with 4% paraformaldehyde. The coverslips were then blocked in 1% bovine serum albumin (BSA) with 0.2% Triton X-100 in phosphate-buffered saline (PBS) for 45 min. Cells were incubated with monoclonal mouse anti-RyR1/2 antibody (Thermo Fisher Scientific, Cat. No. MA3-916, RRID:AB_2183054) or rabbit anti-RyR3 antibody (Alomone Labs, Cat. No. ARR-003, RRID:AB_2040186) at a 1:50 or 1:100 dilution overnight at 4°C. Secondary antibodies conjugated either to Cy2 (1:100) or Cy3 (1:150) (Jackson ImmunoResearch Laboratories, West Grove, PA) were incubated for 1.5 h at room temperature. Incubation with no primary antibody was used as negative control. IF microscopy was also used to confirm the identity of cultured CGCs using the goblet cell-specific lectin Ulex europaeus agglutinin (UEA)-1 and anti-cytokeratin 7 (CK7) antibody (Santa Cruz Biotechnology, CA) (Supplemental Fig. S1) (34).

### Live/Dead Assay

Live/dead assay was performed according to the manufacturer’s instructions (35). First-passage rat CGCs were grown on coverslips overnight. Cells were washed twice with Dulbecco’s phosphate-buffered saline (PBS) before addition of 100% ethanol, PBS alone, or dantrolene 10^−3^ M for 15 min, 30 min, or 45 min. The cells were rinsed again with PBS to remove agents, and the live/dead viability assay was performed. Calcein/AM, which is converted to fluorescent calcein in live cells, and ethidium homodimer-1 (EH-1), which penetrates damaged cell membranes of dead cells, were used to identify live and dead cells. Cells were incubated in a solution containing 2% calcein/AM and 2% EH-1 in PBS for 10 min at room temperature. Cells were viewed by immunofluorescence microscopy, and micrographs were taken with a digital camera. ImageJ software was used to add scalebars to micrographs (36).

### Measurement of [Ca^2+^]_i_

Measurement of [Ca^2+^]_i_ was conducted as previously reported (33). First-passage CGCs were cultured overnight at 37°C on 35-mm glass bottom dishes. CGCs were then incubated in KRB-HEPES supplemented with 0.5% BSA, 0.5 μM Fura-2/AM (Invitrogen, Grand Island, NY), 8 μM Pluronic F-127 (Sigma-Aldrich, St. Louis, MO), and 250 μM sulfinpyrazone (Sigma-Aldrich) at room temperature for 1 h in the dark. Cells were washed with KRB-HEPES containing 250 μM sulfinpyrazone before [Ca^2+^]_i_ measurements. All [Ca^2+^]_i_ measurements were conducted with a fluorescent ratio imaging system (In Cyt Im2; Intracellular Imaging, Cincinnati, OH) (Supplemental Fig. S2). Excitation wavelengths of 340 and 380 nm were used and an emission wavelength of 505 nm was measured allowing the computation of ratiometric values. CGCs were exposed to agonists, antagonists, inhibitors, and chelators under different buffer conditions. The addition of agonists to CGCs was randomized using simple randomization. [Ca^2+^]_i_ was measured for 2–3 min, and the peak [Ca^2+^]_i_ was calculated by subtracting the average of the basal value from the maximal [Ca^2+^]_i_ value. Approximately 20 CGCs were selected for [Ca^2+^]_i_ measurements in each technical replicate, and individual cells with a baseline of more than 500 nM were excluded from further analyses. ATP at 10^−5^ M and BzATP at 10^−4^ M were used throughout this study, as these concentrations of the agonists have been shown to elicit the highest peak [Ca^2+^]_i_ in cultured rat CGCs (32).

ATP, BzATP, thapsigargin, BAPTA/AM, histamine, and carbachol were purchased from Sigma-Aldrich (St. Louis, MO). Nifedipine, U73122, and U73343 were obtained from Tocris (Minneapolis, MN), and dantrolene from Abcam (Cambridge, MA). VIP was purchased from EMD Chemicals (Rockville, MA), and H89 was purchased from R&D Systems (Minneapolis, MN).

### Quantitative Reverse Transcription Polymerase Chain Reaction

Quantitative reverse transcription polymerase chain reaction (RT-qPCR) was performed as previously reported, with minor modifications (33). Total RNA from cultured human and rat CGC was extracted and purified according to the quick-start protocol for miRNeasy Mini kit (Qiagen, CA). NanoDrop 2000 spectrophotometer (Thermo Fisher Scientific, Waltham, MA) was used to determine total RNA concentration. cDNA was synthesized by reverse transcription from 1 µg of RNA using the iScript cDNA synthesis kit (Bio-Rad Laboratories, Inc., Hercules, CA). cDNA was quantified by qPCR using iTaq Universal SYBR Green Supermix (Bio-Rad Laboratories, Inc., Hercules, CA). Absolute copy number of the gene was calculated using the equation (2^(−ΔCt)^) × 1,000,000 and expressed as copy number per one million copies of GAPDH (reference gene). Primer sequences for RT-qPCR were obtained from the PrimerBank (https://pga.mgh.harvard.edu/primerbank/) and are shown in Supplemental Table S1. The specificity of the primers was confirmed using NCBI’s Primer BLAST (https://www.ncbi.nlm.nih.gov/tools/primer-blast/).

### Statistical Analysis

All experiments were performed in triplicate from at least three individuals (Supplemental Fig. S3). Data are expressed as means ± SE and analyzed using Student’s *t* test for two group comparisons. *P* < 0.05 was considered to indicate statistical significance.

## RESULTS

### ATP and BzATP Increase [Ca^2+^]_i_ in Cultured Rat CGCs

**Figure 1.**
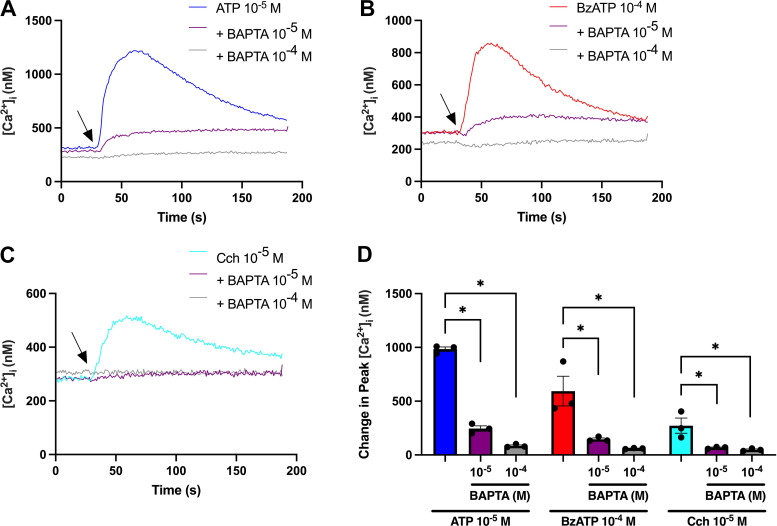
Effect of chelating Ca^2+^_i_ on ATP and BzATP-induced peak [Ca^2+^]_i_. Cultured rat conjunctival goblet cells were preincubated with or without the Ca^2+^_i_ chelator BAPTA/AM (10^−5^ or 10^−4^ M) for 30 min before stimulation with ATP (10^−5^ M), BzATP (10^−4^ M), or the cholinergic agonist, carbachol (Cch, 10^−5^ M), the positive control. *A–C*: the average [Ca^2+^]_i_ over time. Arrows represent the addition of ATP (*A*), BzATP (*B*), or Cch (*C*). *D*: the peak increase of [Ca^2+^]_i_ above baseline. Blue-, red-, and cyan-colored bars represent ATP, BzATP, and Cch alone; purple bars and gray bars represent preincubation with BAPTA/AM 10^−5^ or 10^−4^ M, respectively. Data are presented as means ± SE. *n* = 3. BzATP, benzoylbenzoyl-ATP; [Ca^2+^]_i_, intracellular calcium concentration. *Statistically significant difference from agonist alone.

The Ca^2+^_i_ chelator, BAPTA/AM, was used to show that ATP and BzATP increase [Ca^2+^]_i_ in cultured rat CGCs. Cells were incubated with or without BAPTA/AM (10^−5^ or 10^−4^ M) for 30 min before stimulation with ATP (10^−5^ M), BzATP (10^−4^ M), or the positive control carbachol (Cch) (10^−5^ M) (Fig. 1). Peak [Ca^2+^]_i_ decreased from 984 ± 20 nM for ATP 10^−5^ M alone to 246 ± 26 and 84 ± 9 nM when preincubated with BAPTA/AM 10^−5^ and 10^−4^ M, respectively (Fig. 1*A*). These values were 593 ± 138 nM for BzATP 10^−4^ M alone, which decreased to 146 ± 12 and 60 ± 3 nM after preincubation with BAPTA/AM 10^−5^ and 10^−4^ M, respectively (Fig. 1*B*). Cch-induced peak [Ca^2+^]_i_ decreased from 272 ± 71 nM for Cch 10^−5^ M alone to 68 ± 5 and 49 ± 7 nM with BAPTA/AM 10^−5^ and 10^−4^ M, respectively (Fig. 1*C*). Figure 1*D* shows that ATP, BzATP, and carbachol significantly increased [Ca^2+^]_i_ in cultured rat CGCs and this was blocked by chelation of intracellular Ca^2+^.

### ATP-Induced Peak [Ca^2+^]_i_ Relies on Extracellular Ca^2+^ (Ca^2+^_o_) and Intracellular Calcium Stores

**Figure 2.**
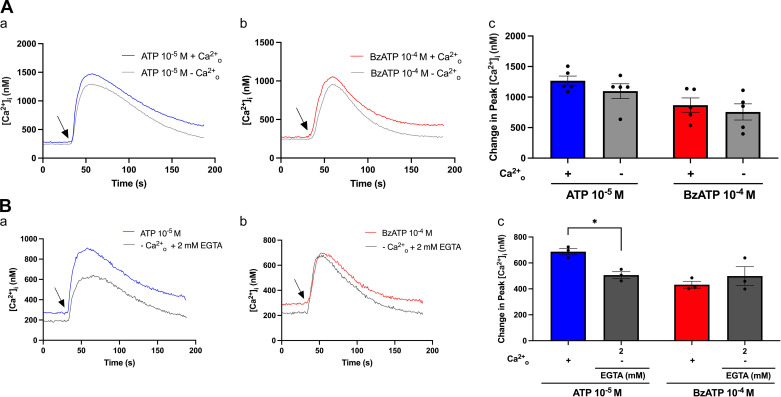
Role of external Ca^2+^ in ATP and BzATP stimulation of [Ca^2+^]_i_. *A*: the effect of omitting extracellular calcium ions (Ca^2+^_o_) on peak [Ca^2+^]_i_ in cultured rat conjunctival goblet cells (CGCs). CGCs were incubated in KRB-HEPES with (+) or without (−) Ca^2+^ before stimulation with ATP (10^−5^ M) or BzATP (10^−4^ M). *A*, *a* and *b*: the average [Ca^2+^]_i_ over time. Arrows represent the addition of ATP (*Aa*) or BzATP (*A*, *b*). *Ac*: the peak increase of [Ca^2+^]_i_ above baseline. Blue and red bars represent buffer conditions with added Ca^2+^, light gray bars represent buffer conditions without added Ca^2+^. *B*: the effect of omitting and chelating Ca^2+^_o_ on peak [Ca^2+^]_i_ in CGCs. CGCs were incubated in KRB-HEPES with (+), or without (−) Ca^2+^_o_ and with EGTA (2 mM) before stimulation with ATP (10^−5^ M) or BzATP (10^−4^ M). *B*, *a* and *b*: the average [Ca^2+^]_i_ over time. Arrows represent the addition of ATP (*Ba*) or BzATP (*Bb*). *Bc*: the peak increase of [Ca^2+^]_i_ above baseline. Dark gray bars represent ATP or BzATP addition to buffer without (−) Ca^2+^_o_ plus EGTA (2 mM). Data are presented as means ± SE (*n* = 5 for *A*; *n* = 3 for *B*). BzATP, benzoylbenzoyl-ATP; [Ca^2+^]_i_, intracellular calcium concentration. *Statistically significant difference from agonist addition to CGCs in buffer containing Ca^2+^ without EGTA supplementation.

Omitting Ca^2+^_o_ can differentiate Ca^2+^ influx through the plasma membrane from the release of Ca^2+^ from internal stores (37). Excluding Ca^2+^ from the KRB-HEPES buffer alone did not significantly decrease ATP- (10^−6^–10^−4^ M) or BzATP (10^−4^ M) -induced peak [Ca^2+^]_i_ (Fig. 2*A*, *a*–*c*). However, ATP but not BzATP-induced peak [Ca^2+^]_i_ was significantly reduced from 687 ± 25 to 506 ± 26 nM when omitting and chelating residual Ca^2+^_o_ with 2 mM EGTA (Fig. 2*B*, *a*–*c*). These results indicate that both Ca^2+^_o_ and Ca^2+^ from internal stores increase [Ca^2+^]_i_ in response to ATP stimulation, but internal stores appear to be more important, as Ca^2+^_o_ only accounts for a small portion of the resulting [Ca^2+^]_i_ increase (Fig. 2*B*, *a*–*c*). However, only internal Ca^2+^ stores seem to be mobilized in response to BzATP addition to cultured rat CGCs.

### Depletion of Intracellular Ca^2+^ Stores by Thapsigargin Reduces the Peak [Ca^2+^]_i_ Elicited by ATP and BzATP

**Figure 3.**
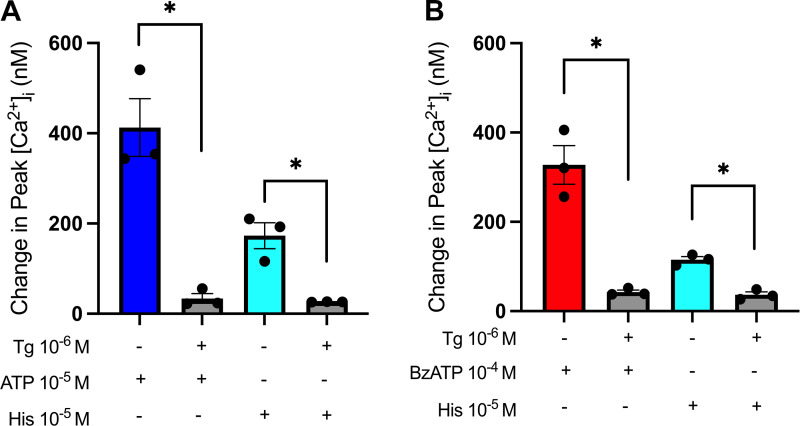
Effect of depleting endoplasmic reticulum Ca^2+^ on ATP and BzATP-induced peak [Ca^2+^]_i_. Cultured rat conjunctival goblet cells were preincubated with thapsigargin (Tg) (10^−6^ M) for 15 min before stimulation with ATP (10^−5^ M) (*A*) or BzATP (10^−4^ M) (*B*). Histamine (His) was used as a positive control. *A* and *B*: the peak increase of [Ca^2+^]_i_ above baseline. Data are presented as means ± SE. *n* = 3. BzATP, benzoylbenzoyl-ATP; [Ca^2+^]_i_, intracellular calcium concentration. *Statistically significant difference from agonist alone.

The sarco/ER Ca^2+^-ATPase (SERCA) inhibitor thapsigargin depletes ER calcium stores (37–40). To investigate whether the ER was the source of intracellular Ca^2+^, CGCs were preincubated with thapsigargin (10^−6^ M) for 15 min before stimulation with ATP (10^−5^ M), BzATP (10^−4^ M), or the positive control histamine (10^−5^ M), and [Ca^2+^]_i_ was measured (Fig. 3). Preincubation with thapsigargin significantly reduced peak [Ca^2+^]_i_ from 413 ± 64 to 34 ± 11 nM and 328 ± 43 to 43 ± 4 nM for ATP (10^−5^ M) and BzATP (10^−4^ M), respectively. Histamine-induced peak [Ca^2+^]_i_ decreased from 173 ± 29 nM with histamine 10^−5^ M alone to 26 ± 1 nM with thapsigargin (Fig. 3*A*). These results indicate that ER calcium stores are responsible for the majority of the [Ca^2+^]_i_ increase observed as a result of ATP and BzATP stimulation of cultured rat CGCs.

### Inhibiting Phospholipase C Did Not Reduce ATP or BzATP-Induced Peak [Ca^2+^]_i_

**Figure 4.**
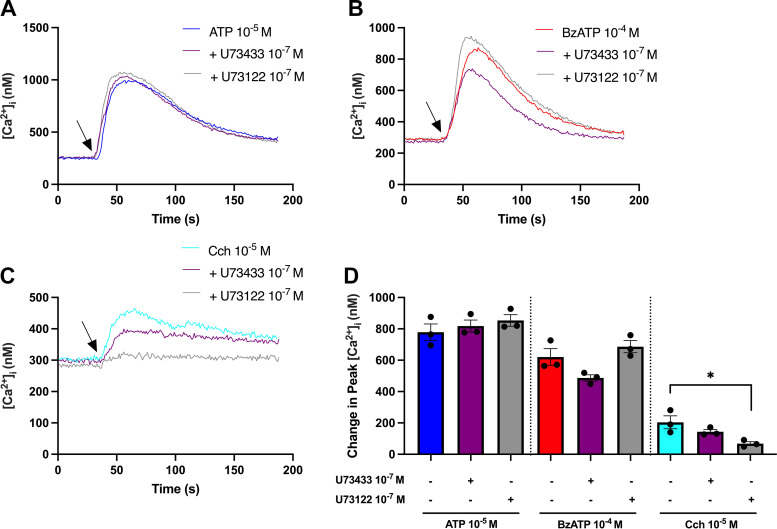
Effect of inhibiting phospholipase C (PLC) on ATP and BzATP-induced peak [Ca^2+^]_i_. Cultured rat conjunctival goblet cells were preincubated with the PLC inhibitor U73122 (10^−7^ M) [gray lines in (*A–C*); gray bars in (*D*)] or the negative control U73433 (10^−7^ M) [purple lines in (*A–C*); purple bars in (*D*)] for 15 min before stimulation with ATP (10^−5^ M), BzATP (10^−4^ M), or carbachol (Cch) (10^−5^ M). Agonists alone are represented by blue (ATP)-, red (BzATP)-, and cyan (Cch)-colored lines and bars in *A–D*. *A–C*: the average [Ca^2+^]_i_ over time. Arrows represent the addition of ATP (*A*), BzATP (*B*), or Cch (*C*). *D:* the peak increase of [Ca^2+^]_i_ above baseline. Cch was used as a positive control. Data are presented as means ± SE. *n* = 3. BzATP, benzoylbenzoyl-ATP; [Ca^2+^]_i_, intracellular calcium concentration. *Statistically significant difference from agonist alone.

P2YRs primarily activate the Gq-PLC pathway (41). Activation of PLC increases IP3, which can thereby activate ER IP3 receptors to release Ca^2+^ (42, 43). To investigate whether ATP and BzATP use the Gq-PLC pathway, rat CGCs were preincubated with the PLC inhibitor U73122 (10^−7^ M) or the negative control U73433 (10^−7^ M) for 15 min, before ATP (10^−5^ M) or BzATP (10^−4^ M) stimulation, and [Ca^2+^]_i_ was measured (Fig. 4). No statistically significant alterations in peak [Ca^2+^]_i_ were observed when stimulating CGCs with ATP or BzATP alone and after inclusion of the active or inactive PLC inhibitor (Fig. 4, *A*–*C*). Cch, the positive control, induced a peak [Ca^2+^]_i_ of 204 ± 41 nM alone, 144 ± 14 nM with U73433, and was significantly reduced to 68 ± 12 nM in the presence of U73122 (Fig. 4*D*). These results indicate that ATP and BzATP are unlikely to use P2YRs to activate IP3 receptors through the PLC pathway to elevate [Ca^2+^]_i_ in rat CGCs.

### Blocking Protein Kinase A Significantly Reduced ATP-Induced Peak [Ca^2+^]_i_

**Figure 5.**
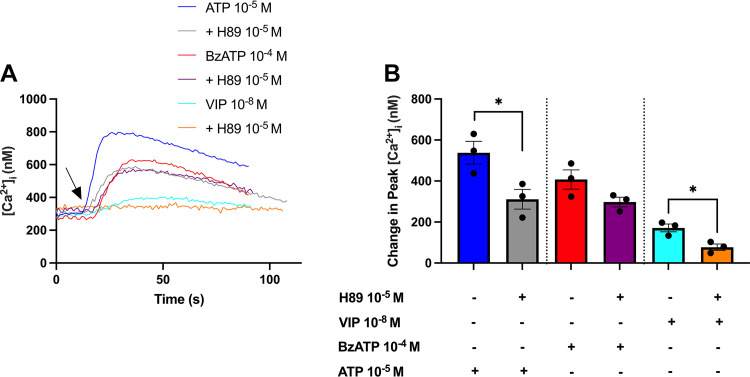
Effect of inhibiting protein kinase A (PKA) on ATP and BzATP-induced peak [Ca^2+^]_i_. Cultured rat CGCs were preincubated with the PKA inhibitor H89 (10^−5^ M) for 30 min before stimulation with ATP (10^−5^ M), BzATP (10^−4^ M), or the positive control, vasoactive intestinal peptide (VIP) (10^−8^ M). *A*: the average [Ca^2+^]_i_ over time. Arrow represents the addition of agonists. *B*: the peak increase of [Ca^2+^]_i_ above baseline. Data are presented as means ± SE. *n* = 3. BzATP, benzoylbenzoyl-ATP; [Ca^2+^]_i_, intracellular calcium concentration; CGCs, conjunctival goblet cells. *Statistically significant difference from agonist alone.

The PKA pathway is used by rat CGCs to increase [Ca^2+^]_i_, and is a major pathway used by P1 receptors (26, 40, 44). To investigate whether ATP and BzATP use the PKA pathway to increase [Ca^2+^]_i_, rat CGCs were preincubated with the PKA inhibitor H89 (10^−5^ M) for 30 min, before ATP (10^−5^ M) or BzATP (10^−4^ M) stimulation, and [Ca^2+^]_i_ was measured (Fig. 5). Peak [Ca^2+^]_i_ was significantly decreased from 538 ± 56 nM for ATP alone to 311 ± 48 nM in the presence of H89. BzATP-induced peak [Ca^2+^]_i_ decreased from 407 ± 47 to 298 ± 24 nM, although this decrease was not statistically significant (*P* = 0.10). For the positive control, vasoactive intestinal peptide (VIP) (10^−8^ M), peak [Ca^2+^]_i_ significantly decreased from 171 ± 19 to 77 ± 16 nM. These results suggest that ATP, and perhaps BzATP, activate P1 receptors to increase cAMP, PKA-activity, and [Ca^2+^]_i_, but further investigation is needed.

### The P1 Receptor Agonist Adenosine Increases [Ca^2+^]_i_ in Rat CGCs

**Figure 6.**
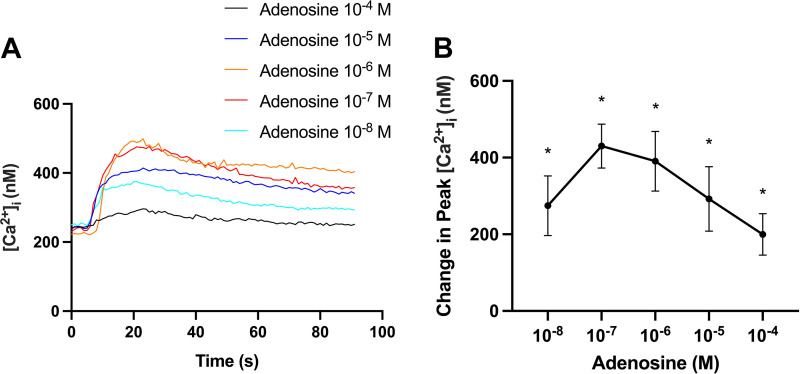
Adenosine increased [Ca^2+^]_i_ in CGCs. CGCs containing Fura-2 were stimulated with adenosine (10^−8^–10^−4^ M), and [Ca^2+^]_i_ was measured. *A*: the average [Ca^2+^]_i_ over time. *B*: the peak increase of [Ca^2+^]_i_ above baseline. Data are shown as means ± SE. *n* = 4. [Ca^2+^]_i_, intracellular calcium concentration; CGCs, conjunctival goblet cells. *Statistically significant difference from baseline.

To evaluate the effect of the nonselective P1 receptor agonist adenosine, rat CGCs containing Fura-2 were stimulated with adenosine (10^−8^–10^−4^ M), and [Ca^2+^]_i_ was measured. Adenosine significantly increased peak [Ca^2+^]_i_ in a dose-dependent manner from 275 ± 78 nM at adenosine 10^−8^ M to a maximum of 430 ± 57 nM at adenosine 10^−7^ M, and then declining to 391 ± 78 nM for adenosine at 10^−6^ M, 292 ± 84 nM for adenosine 10^−5^ M, and 200 ± 54 nM for adenosine at 10^−4^ M (Fig. 6). These data indicate that one or more P1 receptors are functional in rat CGCs.

### CGCs Use the L-Type Calcium Channel and Ryanodine Receptor to Increase [Ca^2+^]_i_ in Response to ATP and BzATP-Stimulation

**Figure 7.**
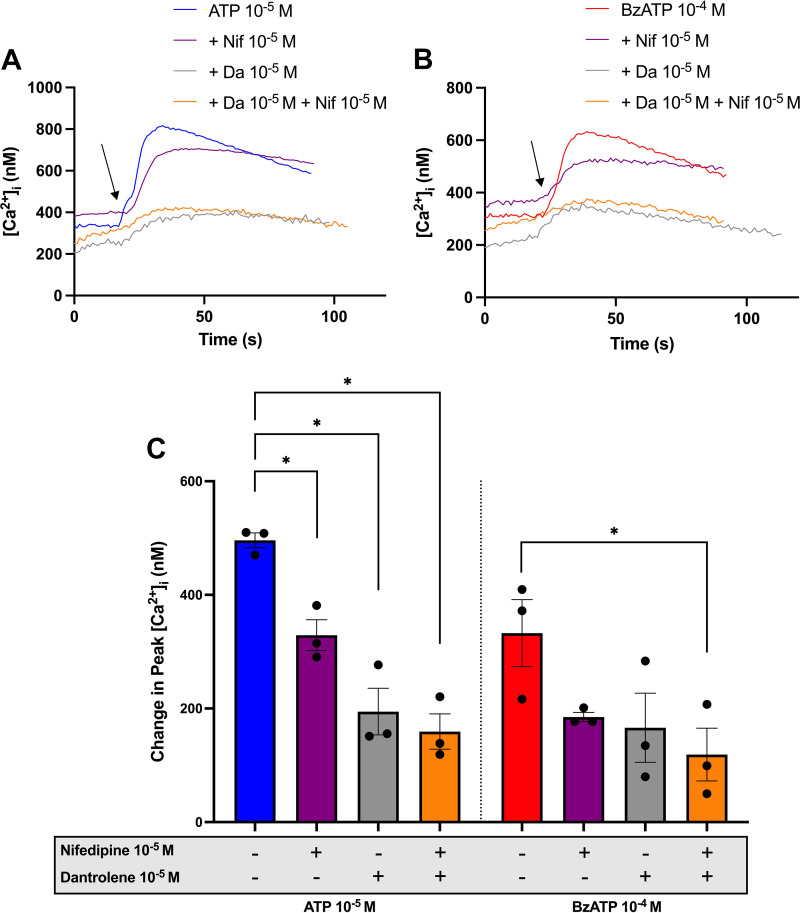
Effect of blocking L-type calcium channels with nifedipine and ryanodine receptors with dantrolene on ATP and BzATP-induced peak [Ca^2+^]_i_. Cultured rat conjunctival goblet cells were preincubated with nifedipine (Nif) (10^−5^ M) for 15 min and/or dantrolene (Da) (10^−5^ M) for 30 min before stimulation with ATP (10^−5^ M) (*A*) or BzATP (10^−4^ M) (*B*). *A* and *B*: the average [Ca^2+^]_i_ over time. Arrows represent the addition of agonists. *C*: the peak increase of [Ca^2+^]_i_ above baseline. ATP: bars to the left of the central vertical dotted line in *C*, BzATP: bars to the right of the central vertical dotted line in *C*. Data are presented as means ± SE. *n* = 3. BzATP, benzoylbenzoyl-ATP; [Ca^2+^]_i_, intracellular calcium concentration. *Statistically significant difference from agonist alone.

Voltage-gated calcium channels (VGCCs), such as the L-type calcium channel, are functional in CGCs (45–47). They are activated by membrane depolarization and antagonized by nifedipine (48). The VGCCs often function in a smooth interplay with RyRs in excitable cells, and both VGCCs and RyRs are positively modulated by PKA (49). Dantrolene is a RyR antagonist (50). To investigate whether the L-type calcium channel or RyRs are involved in the [Ca^2+^]_i_ increase elicited by ATP and BzATP, CGCs were preincubated with nifedipine (10^−5^ M) for 15 min and/or dantrolene (10^−5^ M) for 30 min, before ATP (10^−5^ M) or BzATP (10^−4^ M) addition, and [Ca^2+^]_i_ was measured (Fig. 7). Both nifedipine and dantrolene significantly reduced peak [Ca^2+^]_i_ induced by ATP and BzATP. Peak [Ca^2+^]_i_ decreased significantly from 496 ± 13 nM for ATP alone to 329 ± 27, 195 ± 41, and 159 ± 31 nM in the presence of nifedipine, dantrolene, or their combination, respectively. BzATP-induced peak [Ca^2+^]_i_ decreased from 333 ± 59 to 185 ± 8 (*P* = 0.068), 166 ± 61 (*P* = 0.12), and 119 ± 46 nM (*P* = 0.046), respectively. These results indicate that ATP and BzATP increase [Ca^2+^]_i_ by activating the L-type calcium channel and RyRs.

### Omitting Extracellular Magnesium (Mg^2+^_o_) Potentiates BzATP-Induced Peak [Ca^2+^]_i_

**Figure 8.**
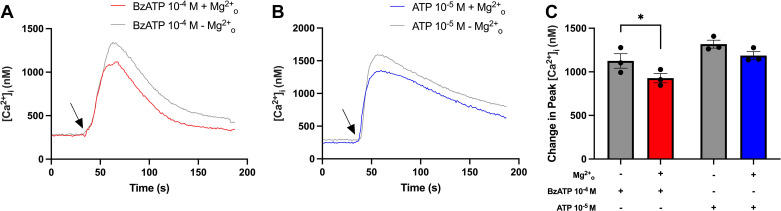
Effect of Mg^2+^_o_ on peak [Ca^2+^]_i_ in cultured rat conjunctival goblet cells (CGCs). CGCs incubated with Fura-2/AM were stimulated with ATP (10^−5^ M) or BzATP (10^−4^ M) in KRB-HEPES without (gray traces; gray bars) or with Mg^2+^_o_ (red or blue traces; red or blue bars). The average [Ca^2+^]_i_ level for three rats over time is shown in *A* and *B*. Arrows represent the addition of ATP (*A*) or BzATP (*B*). *C*: the peak increase of [Ca^2+^]_i_ above baseline. Data are shown as means ± SE. *n* = 3. BzATP, benzoylbenzoyl-ATP; [Ca^2+^]_i_, intracellular calcium concentration. *Statistically significant difference from grey bar.

RyRs are known to be negatively modulated in the presence of Mg^2+^_o_ (15–17). Therefore, ATP (10^−5^ M) or BzATP (10^−4^ M) was added to rat CGCs in buffer with or without Mg^2+^, after which [Ca^2+^]_i_ was measured. Figure 8, *A* and *B*, shows the average [Ca^2+^]_i_ over time. As shown in Fig. 8*C*, peak [Ca^2+^]_i_ significantly decreased from 1,125 ± 83 nM to 928 ± 53 nM in response to BzATP without or with Mg^2+^_o_, respectively. The addition of ATP after preincubating CGCs without or with Mg^2+^_o_ was not significantly altered but decreased from 1,263 ± 46 to 1,187 ± 45 nM. These results can indicate that RyRs are activated by BzATP, and possibly ATP, in cultured rat CGCs.

### Ryanodine Receptor 3 Is Present in Rat and Human CGCs

**Figure 9.**
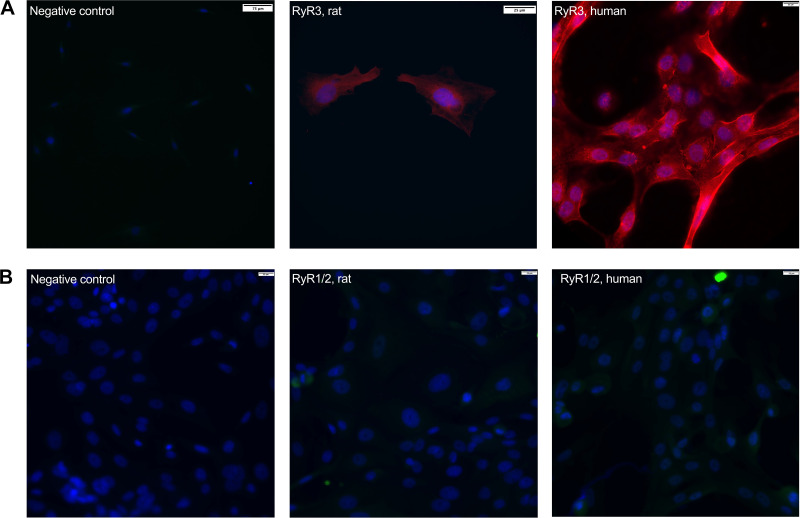
Identification of ryanodine receptors (RyRs) in rat and human conjunctival goblet cells (CGCs). Immunofluorescence microscopy was used to determine the presence of RyR3 (*A*) or RyR1/2 (*B*) from rat (*middle*) and human (*right*) CGCs. Negative controls (*left*) are representative for rat and human cells. Red: RyR3, green: RyR1/2, blue: DAPI. Images are representative for at least three individuals. Scale bars are located in the upper right corner of all micrographs. Scale bar = 25–75 μm.

To identify the RyR isoforms present in CGCs, CGCs were stained with specific RyR1/2 or RyR3 antibodies. Positive CGC staining was found for RyR3 in both rat and human CGCs (Fig. 9*A*) but not for RyR1 or 2 isoforms in either species (Fig. 9*B*).

### RT-qPCR Confirmed the Expression of Ryanodine Receptor 3 in CGCs

**Figure 10.**
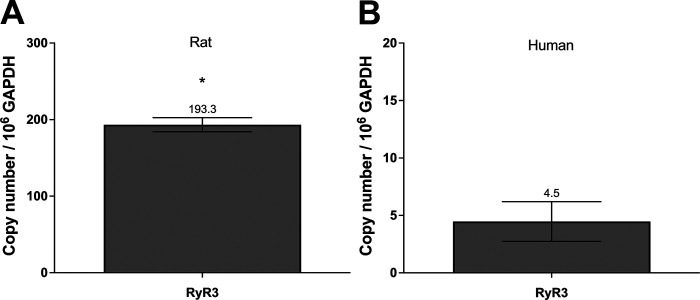
RT-qPCR of the ryanodine receptor 3 (RyR3) subtype in rat and human conjunctival goblet cells (CGCs). Total RNA was isolated from rat and human CGCs and reverse-transcribed. cDNA amplification confirmed the presence of RyR3 mRNA in rat (*A*) but not in human (*B*) CGCs. Relative amount of RyR3 transcript compared with glyceraldehyde-3-phosphate dehydrogenase (GAPDH) was measured by quantitative PCR and is shown on *y*-axis. *n* = 3. RT-qPCR, quantitative reverse transcription polymerase chain reaction. *Statistically significant difference from 0.

To determine whether mRNA of the RyR3 isoform is expressed in CGCs, RT-qPCR was performed on RyR3 RNA from rat and human CGCs (Fig. 10). This confirmed the presence of RyR3 mRNA compared with the housekeeping gene glyceraldehyde-3-phosphate dehydrogenase (GAPDH) in rat (*P* < 0.001) but not in human (*P* = 0.06) CGCs. These results further implicate a role for the RyR3 isoform in rat, and perhaps human, CGCs.

### Dantrolene Reduces Baseline [Ca^2+^]_i_ in Rat CGCs

**Figure 11.**
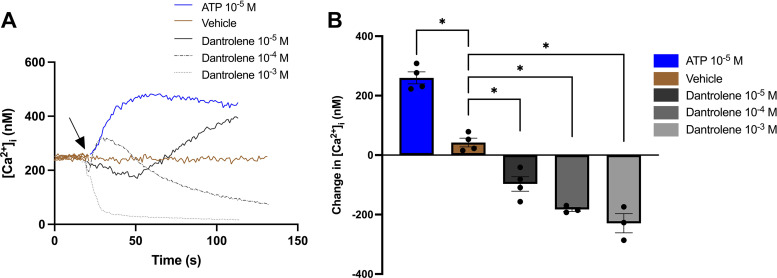
The effect of dantrolene on [Ca^2+^]_i_ in rat conjunctival goblet cells (CGCs). Fura-2 containing CGCs was stimulated with dantrolene (10^−5^–10^−3^ M) or ATP (10^−5^ M) during [Ca^2+^]_i_ measurement. *A*: the average of 3 rats and shows [Ca^2+^]_i_ over time. *B*: the maximal change of [Ca^2+^]_i_ from baseline. Note that the bars for dantrolene show the maximal negative changes of [Ca^2+^]_i_ from baseline. Arrow represents the addition of compounds or vehicle. Data are presented as means ± SE. *n* = 3. [Ca^2+^]_i_, intracellular calcium concentration. *Statistically significant difference from vehicle.

To investigate the effect of the RyR antagonist dantrolene on baseline [Ca^2+^]_i_, CGCs were incubated in KRB-HEPES before the addition of dantrolene (10^−5^–10^−3^ M) and [Ca^2+^]_i_ measurement. As shown in Fig. 11*A*, ATP alone increased [Ca^2+^]_i_ to 260 ± 20 nM. After initially declining, [Ca^2+^]_i_ increased ∼50 s after dantrolene 10^−5^ M application. After the application of dantrolene 10^−4^ M, [Ca^2+^]_i_ first increased before declining below baseline. Dantrolene (10^−3^ M) reduced [Ca^2+^]_i_ with 229 ± 32 nM from baseline to almost 0 nM. These results indicate that dantrolene alone blocks the RyR3 and decreases the [Ca^2+^]_i_ resulting in a net flux of Ca^2+^ into the ER by the SERCA Ca^2+^-ATPase. Thus, the normal regulation of [Ca^2+^]_i_ is disrupted. In addition, dantrolene blocked ATP-stimulated increase in [Ca^2+^]_i_ as shown in Fig. 7 Taken together, these results suggest that ATP increases [Ca^2+^]_i_ through the activation of RyR3 in cultured rat CGCs.

### High Concentrations of the Ryanodine Receptor Antagonist Dantrolene Induce Cell Death of CGCs

**Figure 12.**
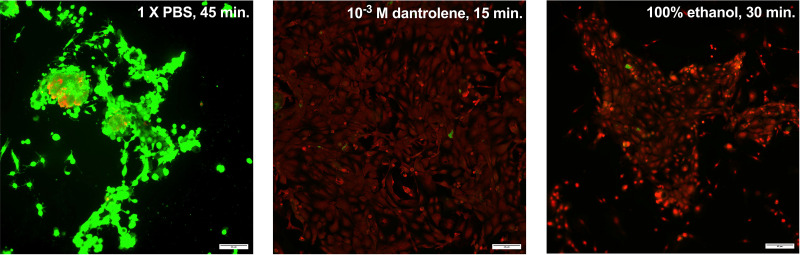
Live/dead assay of cultured rat conjunctival goblet cells treated with 1X PBS, dantrolene 10^−3^ M, or 100% ethanol. Green indicates live cells; red indicates dead cells. Images are representative of 3 rats. Scale bars are located in the lower right corner of all micrographs. Scale bar = 45 μm.

To investigate whether the large reduction in [Ca^2+^]_i_ observed after the application of 10^−3^ M dantrolene leads to cell death of the CGCs, a live/dead assay was performed on CGCs treated with 1X PBS for 45 min (left), 10^−3^ M dantrolene for 15 min (center), or 100% ethanol for 30 min (right). As shown in Fig. 12, the application of dantrolene 10^−3^ M for 15 min killed the majority of the CGCs. This indicates that blocking the RyR3 leads to disruption of Ca^2+^_i_ homeostasis and cell death of cultured rat CGCs.

## DISCUSSION

**Figure 13.**
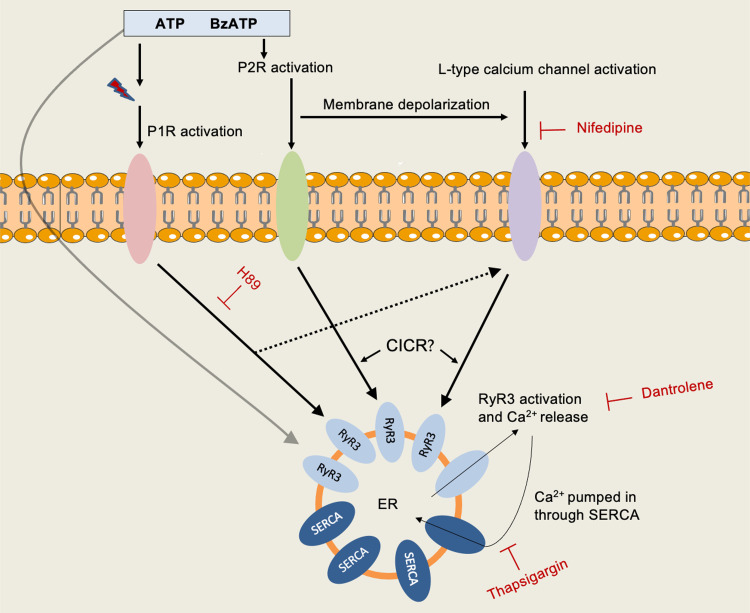
Proposed regulation of intracellular calcium levels by ryanodine receptor 3 (RyR3) in cultured rat conjunctival goblet cells. ATP and BzATP may increase [Ca^2+^]_i_: *1*) directly by activating RyR3, *2*) by activating purinergic 1 (P1) receptors after the enzymatic conversion to metabolites, *3*) by the activation of purinergic 2 (P2) receptors directly, and *4*) by activating L-type calcium channels through membrane depolarization. These mechanisms can then either directly increase [Ca^2+^]_i_ through the influx of extracellular Ca^2+^ and/or through the activation of RyR3 on an intracellular compartment such as the ER to release Ca^2+^. BzATP, benzoylbenzoyl-ATP; [Ca^2+^]_i_, intracellular calcium concentration; CGCs, conjunctival goblet cells; CICR, calcium-induced calcium release.

ATP is released during inflammation of the ocular surface and probably also functions in normal physiology of goblet cell mucous stimulation (51–53). Both ATP and its analog BzATP stimulate mucin secretion in rat CGCs in vitro (32). Herein, investigation of the source of Ca^2+^ mobilized by ATP and BzATP found multiple signaling pathways in rat CGCs (Fig. 13). First, ATP and BzATP increased peak [Ca^2+^]_i_ even when Ca^2+^_o_ was omitted from the buffer, suggesting mobilization of Ca^2+^ from intracellular stores. This indicates that ATP and BzATP are not solely using P2X receptors that are ionotropic plasma membrane-bound channels (54). Even when chelating Ca^2+^_o_, [Ca^2+^]_i_ rose to a high level after stimulation with the two agonists, with a plateau phase resembling that seen after the stimulation of hepatocytes with the RyR agonist cADPR (55, 56). Only ATP showed a statistically significant decrease in peak [Ca^2+^]_i_ after omitting Ca^2+^_o_ from the buffer plus chelating any residual Ca^2+^_o_ present. Similar findings are reported in other cell types, where ATP is dependent on both extracellular and intracellular calcium stores to increase [Ca^2+^]_i_ (39, 57, 58). A point to note is that EGTA has been reported to chelate Ca^2+^_i_ in the presence of a high, membrane permeabilizing concentration of ATP (59).

Second, ATP and BzATP used ER calcium stores to increase [Ca^2+^]_i_ based on experiments with thapsigargin, but did not activate PLC. P2Y receptors, another form of the P2 receptors that are known to be activated by ATP and BzATP, are GPCRs that in epithelial cells activate PLC to produce IP3 and stimulate IP3 receptors on the ER to increase [Ca^2+^]_i_ (41, 60). Previous studies on rabbit CGCs suggested that the P2Y2R is responsible for ATP-induced mucin release, although the P2Y2 and P2Y1R were the only purinergic receptors investigated to our knowledge, with the use of the nonspecific antagonists suramin and PPADS (61–63). We did not find evidence for P2YR activation by ATP or BzATP through the Gq-PLC pathway.

Third, we found that adenosine likely activated P1 receptors to increase [Ca^2+^]_i_. ATP, and possibly BzATP, can be metabolized by ectonucleotidases into adenosine that stimulates P1 receptors (27, 64). Activation of P1 receptors can in turn stimulate PKA to increase [Ca^2+^]_i_ (30, 65). The increase in [Ca^2+^]_i_ observed after adenosine stimulation indicates that the P1 receptors include the cAMP-inducing A2-group of adenosine receptors (A2AR) (66). In addition, adenosine stimulated a large, dose-dependent [Ca^2+^]_i_ increase in our CGCs. Little is known about adenosine receptors in conjunctival epithelial cells; however, the A2 group of adenosine receptors is expressed in the airway epithelium as well as in intestinal mucosal epithelial cells (67–70). Based on affinity data for adenosine receptors in rat and our adenosine dose-response experiment, it is most likely that the cAMP-increasing A2AAR, and perhaps the cAMP-reducing A3AR, are activated by adenosine in cultured rat CGCs (66). To confirm these findings, future studies should investigate the presence of ectonucleotidases on CGCs and use specific P1 antagonists before ATP and BzATP-stimulation.

Finally, the [Ca^2+^]_i_ increase elicited by ATP and BzATP appears to be due to Ca^2+^ influx via L-type calcium channels and RyR3 activation to release ER Ca^2+^ stores as shown by the effects of nifedipine and dantrolene. ATP and BzATP could activate the L-type calcium channel and RyR3 through phosphorylation by PKA in rat CGCs (49, 71). IP3 receptors may function in a synergistic interplay with RyR3 to stabilize [Ca^2+^]_i_, as proposed in other nonexcitable cell types, although IP3 is considered to be a necessity for IP3 receptor activation (56, 72–74). However, a study in human tracheal epithelial cells found that the [Ca^2+^]_i_ increase was induced by RyR, but not IP3 receptor activation (75). Future studies should use 2-APB or another antagonist of IP3 receptors to investigate the relationship between IP3 receptors and RyRs in CGCs. In rat CGCs, the ATP analog 2MeSATP activates P2X4 receptors to increase peak [Ca^2+^]_i_ and induce mucin secretion (32). A cation influx following activation of ionotropic P2X receptors can stimulate VGCCs through membrane depolarization to increase [Ca^2+^]_i_ (46, 76). RyR3 also appears to be crucial for the maintenance of Ca^2+^_i_ homeostasis in CGCs, as disruption of RyR3 function with a high concentration of dantrolene led to a precipitous decrease in [Ca^2+^]_i_ and cell death. A mechanistic hypothesis for CGC death after large dantrolene exposure is ER Ca^2+^ overload with a subsequent supranormal Ca^2+^ transfer from the ER to the cytoplasm and mitochondria (77, 78). It is possible that RyR inactivity may result in excessive ER calcium with a following reduction of store-operated Ca^2+^ entry, which is observed in our CGCs as a reduced [Ca^2+^]_i_ (13, 79). To further investigate this hypothesis, future studies should investigate ER Ca^2+^ concentration after dantrolene exposure of CGCs (80). Evidence of RyRs’ important roles in normal physiology and in the pathophysiology of several life-threatening acute and chronic diseases has emerged in both excitable and nonexcitable cells. RyRs most likely play significant roles in a variety of functions and pathological conditions, such as in neurodegeneration (19, 81–83), epilepsy (50, 84), heart failure and associated skeletal muscle function impairment (16, 85, 86), insulin regulation (87), and epithelial function (88–91).

Calcium-induced calcium release (CICR) is a feature of RyRs and a hallmark of excitable muscle and nerve cells, but RyRs and CICR are also found in nonexcitable cells such as in the rat parotid gland and bovine corneal epithelial cells (91, 92). In enteroendocrine epithelial cells, [Ca^2+^]_i_ increases from the result of an initial influx of Ca^2+^ through the plasma membrane, followed by CICR through RyR3 activation (93). Likewise, CICR is mediated by PKA-facilitated permeation of L-type calcium channels and RyR2 in pancreatic β-cells (94). CICR is potentiated in the presence of ATP, although at higher concentrations than those used in this study (95). Since ATP is released by damaged cells and is shown to increase [Ca^2+^]_i_ through RyR3 activation in this study, it seems valuable to further study the relationship between ATP in inflammation and RyR function in CG and other cells. A study of sympathetic nerves arising from the superior cervical ganglion showed that RyR3 expression and protein levels decline with advancing age (96). As aging is an important risk factor for many diseases, more research is needed on the impact of RyR3 on calcium homeostasis in cells, such as in goblet cells of the conjunctiva in dry eye disease and senescence (97).

In summary, Ca^2+^_o_ plays a minor role in the increase of [Ca^2+^]_i_ in CGCs after stimulation with ATP and BzATP. These purinergic agonists use ER calcium stores to increase peak [Ca^2+^]_i_. A proportion of ATP and BzATP possibly activates the PKA signaling pathway through P1 and P2 receptors. Furthermore, the [Ca^2+^]_i_ increase elicited by ATP and BzATP is likely a response to L-type calcium channel and RyR3 activation (Fig. 13). RyR3 appears to be crucial for CGCs’ intracellular calcium homeostasis; disruption of RyR3 function with a high concentration of dantrolene led to cell death of rat CGCs in this study.

### Conclusions

We conclude that RyR3 is present and crucial for the homeostasis of [Ca^2+^]_i_ in rat CGCs, as inhibition of RyR3 results in cell death. ATP and BzATP increase [Ca^2+^]_i_ through both extracellular and intracellular Ca^2+^ stores possibly through PKA, L-type calcium channels, and RyR3 activity. Our study highlights the importance of RyR3 in pathology associated with defective calcium signaling in epithelial cells.

## DATA AVAILABILITY

Raw data were generated at Schepens Eye Research Institute. Derived data supporting the findings of this study are available from the corresponding author H.F. upon reasonable request.

## SUPPLEMENTAL MATERIAL

10.6084/m9.figshare.26198333 Supplemental Figs. S1–S3 and Supplemental Table S1: https://doi.org/10.6084/m9.figshare.26198333.

## GRANTS

This research was funded by NFR 271555/F20 grant by the Norwegian Research Council, through The Medical Student Research Program (to H.F. and K.F.), and National Institutes of Health (NIH) Grant R01EY 019470 (to D.D.).

## DISCLOSURES

Tor Paaske Utheim is the cofounder and co-owner of The Norwegian dry eye clinic, which delivers talks for and/or receives financial support from the following: ABIGO, Alcon, Allergan, AMWO, Bausch&Lomb, European school for advanced studies in ophthalmology, InnZ Medical, Medilens Nordic, Medistim, Novartis, Santen, Specsavers, Shire and Thea Laboratories. He has served on the global scientific advisory board for Novartis and Alcon as well as the European advisory board for Shire. Utheim is the Norwegian Global Ambassador for Tear Film and Ocular Surface Society (TFOS) and a Board Member of the International Ocular Surface Society. None of the other authors has any conflicts of interest, financial or otherwise, to disclose.

## AUTHOR CONTRIBUTIONS

H.K.F., K.A.F., and D.D. conceived and designed research; H.K.F., K.A.F., M.Y., O.K.R., J.B., and C.L. performed experiments; H.K.F., K.A.F., M.Y., O.K.R., J.B., and C.L. analyzed data; H.K.F., K.A.F., M.Y., J.B., C.L., and D.D. interpreted results of experiments; H.K.F. and K.A.F. prepared figures; H.K.F. and D.D. drafted manuscript; H.K.F., M.Y., T.P.U., and D.D. edited and revised manuscript; H.K.F., K.A.F., M.Y., O.K.R., J.B., C.L., T.P.U., and D.D. approved final version of manuscript.
